# Healthcare providers’ beliefs about the health effects of nicotine and electronic cigarettes

**DOI:** 10.1371/journal.pone.0330962

**Published:** 2026-06-02

**Authors:** Sarahrose Jonik, Daniel Berger, Shari Hrabovsky, Jonathan Foulds, Jessica Yingst

**Affiliations:** 1 Department of Internal Medicine, Penn State Health Milton S. Hershey Medical Center, Hershey, Pennsylvania, United States of America; 2 Departments of Emergency Medicine and Internal Medicine, Virginia Commonwealth University Health System, Richmond, Virginia, United States of America; 3 Penn State University College of Nursing, Hershey, Pennsylvania, United States of America; 4 Department of Public Health Sciences, Penn State University College of Medicine, Hershey, Pennsylvania, United States of America; Universiti Malaysia Terengganu, MALAYSIA

## Abstract

**Background:**

Previous research has shown that a significant proportion of healthcare providers hold inaccurate beliefs about the direct health risks of nicotine. However, the extent to which these misconceptions impact beliefs about the health effects of electronic cigarettes (e-cigarettes) remains unclear. This study examines the relationship between beliefs about nicotine and e-cigarettes among healthcare providers and trainees.

**Methods:**

A questionnaire was distributed via email and printed flyers to healthcare providers and medical trainees at an academic medical center. Participants rated their beliefs about the health effects of nicotine harms on a Likert scale for 3 statements: “Nicotine is the substance that... 1) makes people want to smoke, 2) causes most of the cancer related to tobacco 3) causes most cardiovascular disease (CVD) associated with smoking.” Participants were also asked, “Compared to smoking cigarettes, would you say that electronic cigarettes are...” (less harmful, just as harmful, more harmful). Means and frequencies were tabulated, and logistic regression identified variables associated with believing e-cigarettes to be less harmful than combustible cigarettes.

**Results:**

Participants (n = 598) were 75.8% female with a mean age of 36.4 years (SD = 13.3). The distribution was as follows: 38.7% (n = 232) were physicians, PAs, or NPs, 27.6% (n = 165) were students, 24.4% (n = 146) were RNs, and 9.2% (n = 55) were RTs. Although 91.5% correctly identified nicotine as the chemical that makes people want to smoke, 25.9% and 42.8% incorrectly believed nicotine is the cause of cancer and CVD associated with cigarette smoking, respectively. Only 21.4% identified e-cigarettes as less harmful than cigarettes. Those believing e-cigarettes to be less harmful than combustible cigarettes were more likely to be male (OR=2.11, 95% CI 1.32–3.37), a student (OR=1.88, 95% CI 1.20–2.94), and to disagree that nicotine is the main substance that causes cancer (OR=1.39, 95% CI 1.03–1.88) or CVD (OR=1.62, 95% CI 1.23–2.13).

**Conclusions:**

Inaccurate beliefs regarding nicotine harms persist among healthcare providers and are associated with beliefs about e-cigarette harms. Targeted education on the distinct risks of nicotine, e-cigarettes, and combustible tobacco products is crucial to improving understanding and to support evidence-based counseling on harm reduction strategies.

## Introduction

Cigarette smoking remains the leading cause of disease, disability, and preventable death across the United States (US), accounting for almost one in five preventable deaths each year [1]. Smoking contributes to significant morbidity and mortality by leading to severe health conditions, including cardiovascular disease (CVD), chronic obstructive pulmonary disease (COPD), malignancy, and reproductive complications. These adverse outcomes stem from the thousands of harmful constituents found in cigarette smoke, such as carbon monoxide, arsenic, benzene, and hydrogen cyanide, in addition to nicotine [2,3].

While nicotine itself is a key driver of tobacco dependence due to its addictive properties [2,4], it is not the principal cause of these smoking-related diseases [5,6]. The pharmacologic effects of pure nicotine result from its activation of nicotinic acetylcholine receptors (nAChRs) in the central nervous system, driving addiction through dopaminergic reward pathways [7]. Nicotine’s acute, transient sympathomimetic effects on the cardiovascular, gastrointestinal, and respiratory systems have been well-documented; however, the definitive long-term implications of pure nicotine exposure remain an area of ongoing study [8–11]. For example, the International Agency for Research on Cancer (IARC) has identified over 500 chemicals as possible human carcinogens, but not nicotine [12].

This distinction emphasizes the importance of understanding harm reduction, which is a framework aimed at reducing overall health risks associated with tobacco use. The goal is to prevent nicotine use among non-tobacco users, while simultaneously acknowledging that non-combustible nicotine products, with or without tobacco, carry a lower risk profile compared to combustible tobacco use [5]. Despite this principle, public and healthcare provider beliefs tend to overestimate nicotine’s role in smoking-related disease, largely due to inadequate information regarding its harms [13,14]. Studies have highlighted the persistence of these misconceptions among both the general public [15–17] and healthcare professionals [13,14,18], who may inaccurately attribute the harms of tobacco products solely to nicotine, rather than to the thousands of other toxicants present in combustible tobacco smoke.

The increasing availability of alternative and potentially less harmful nicotine delivery systems, such as electronic cigarettes (e-cigarettes), oral nicotine pouches, and heated tobacco (IQOS), further complicates these misconceptions [13]. E-cigarettes, for example, deliver nicotine via inhalation of a heated aerosol, rather than via combustion, resulting in an overall significantly lower exposure to harmful chemicals compared to traditional cigarette smoke [13,19,20]. Other studies have further demonstrated that e-cigarettes produce fewer pollutant compound exposures relative to tobacco smoke [21] and appear to overall be less toxic than tobacco smoking [22]. However, e-cigarettes are not without risk, as their composition includes substances such as propylene glycol, vegetable glycerin, various flavorings, solvents, and other known tobacco-related toxicants, some of which may pose potential health concerns [23–25]. Additionally, the World Health Organization (WHO) remains cautious about the role of e-cigarettes due to their uptake by non-smokers and insufficient evidence regarding their long-term safety and effects [26]. Nonetheless, it has been suggested that their short-term use for cessation efforts could merit consideration [27,28].

Healthcare providers play a critical role in guiding patients towards evidence-based harm reduction strategies, but their ability to do so is often hindered by the prevalence of these incorrect beliefs about nicotine and e-cigarette harms, particularly regarding their role in harm reduction. While e-cigarettes may offer a less harmful alternative to combustible tobacco, reluctance to recommend these products is influenced by insufficient data on long-term effects coupled with these persistent misconceptions [29,30]. Addressing these educational gaps and public health misinformation is crucial to equipping healthcare professionals with the knowledge needed to effectively counsel patients on harm reduction strategies.

For these reasons, this study aimed to investigate healthcare providers’ beliefs about nicotine-caused harms, focusing on how these beliefs affect beliefs about the health effects of e-cigarettes. By examining this association, the study sought to fill existing gaps in literature and provide actionable insights to guide the development of evidence-based educational interventions.

## Methods

A questionnaire about beliefs regarding nicotine and e-cigarette harms was distributed to healthcare providers and trainees at the Penn State Health Hershey Medical Center, a tertiary academic medical center in Central Pennsylvania, during the summer of 2022. Recruitment occurred from September 6, 2022, to October 23, 2022, using email and printed flyers containing a link and QR code for participants to access the questions. Data was collected by convenience sampling and managed using REDCap electronic data capture tools hosted at Penn State Health Milton S. Hershey Medical Center and Penn State College of Medicine. REDCap (Research Electronic Data Capture) is a secure, web-based application designed to support data capture for research studies [31]**.**

Participation in this study required meeting three eligibility criteria: (1) being a healthcare provider or healthcare provider trainee at the institution (resident, fellow, attending physician, physician assistant (PA), nurse practitioner (NP), registered nurse (RN), respiratory therapist (RT), medical student); (2) being 18 years of age or older; and (3) having proficiency in English. All participants received a detailed explanation of the study, and informed consent was obtained through their voluntary decision to proceed by clicking to continue with the questionnaire. Participants who completed the questionnaire and chose to provide their email address were entered into a drawing to receive a $25 gift card. The study was approved by the Penn State University Institutional Review Board (#20077).

The questionnaire asked demographic questions including age, gender, profession, smoking history, and prior smoking cessation training (yes/no). Participants were then asked to rate their beliefs about the harms of nicotine on a Likert Scale (strongly agree, agree, neutral, disagree, strongly disagree) through 3 statements: 1) “Nicotine is the main substance in tobacco that makes people want to smoke”, 2) “The nicotine in cigarettes is the substance that causes most of the cancer caused by smoking”, 3) “The nicotine in cigarettes is the substance that causes most of the cardiovascular disease caused by smoking.” Responses to these questions were categorized into 3 groups; agreed (strongly agree, agree), neutral, and disagree (strongly disagree, disagree).

Participants were also asked to rate their belief about e-cigarette harms on a 5-point scale (much less harmful, less harmful, just as harmful, more harmful, much more harmful) through the following question: “New types of cigarettes are now available called electronic cigarettes or e-cigarettes (also known as vape-pens, hookah pens, e-hookahs, or e-vaporizers). These products deliver nicotine through a vapor. Compared to smoking cigarettes, would you say that electronic cigarettes are” … These responses were dichotomized for analysis into “less harmful” (much less harmful or less harmful) and “not less harmful” (just as harmful, more harmful, much more harmful). Questions about nicotine and e-cigarette related harms were adapted from the 2017 Health Information National Trends Survey (HINTS) [32,33].

SAS version 9.4 (SAS Institute Inc., Cary, USA) was utilized for data analysis. Means, with standard deviations, and frequencies were used to describe the sample. T-tests and chi-square analyses were used as appropriate to test for differences in outcomes between provider types and age groups (<25, 25–34, 34–44, 45 + years). A logistic regression model was used to predict variables associated with believing e-cigarettes to be less harmful than combustible cigarettes. Predictor variables included in the model were gender, age, previous smoking cessation training, and tobacco use.

## Results

Participant demographics are summarized in Table 1. Of 598 respondents‌‌, 75.8% (n = 447/590) were female, with a mean age of 36.4 years (SD = 13.3) (n = 589). Healthcare roles included physicians, PAs, or NPs (38.7%), students (27.6%), RNs (24.4%), and RTs (9.2%). Smoking prevalence was low, with 2.6% reporting current use and 14.0% reporting former use. Only 27.4% reported prior smoking cessation training.

**Table 1:** Baseline demographic characteristics of healthcare providers and trainees (n=598).

Characteristic	n (%)
Female	447 (75.8)
Mean age (years)	36.4 (SD = 13.3)
Healthcare provider type	
Physicians/NPs/PAs	232 (38.7)
Students	165 (27.6)
Registered nurses	146 (24.4)
Respiratory therapists	55 (9.2)
Smoking status	
Current smokers	15 (2.6)
Former smokers	84 (14.0)
Never smokers	499 (83.4)
Prior smoking cessation training	164 (27.4)

**Fig 1:**
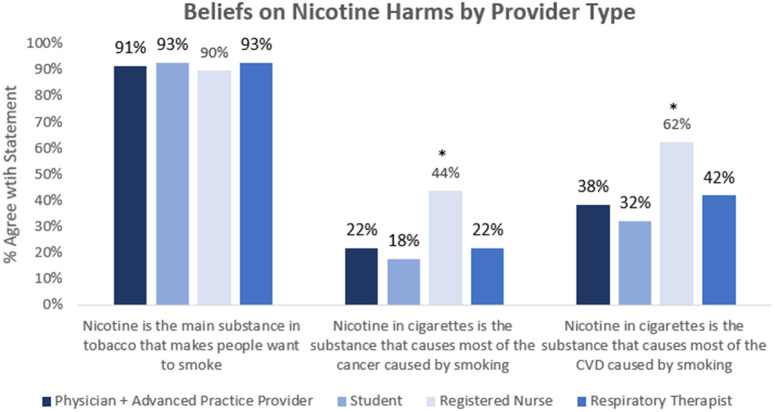
Proportion of participants who agree with each statement by provider type. Graph shows agreement with each statement. Significance marker * denotes that proportion agreeing is significantly higher in this group versus all others, p-value < 0.01.

Overall, the majority (91.5%) agreed that nicotine is the substance in cigarettes that makes people want to smoke. There were no differences in this belief between provider types (p = .80) or age group (p = .47). Fewer (25.9%) believed nicotine in cigarettes to be the substance that causes most of the cancer caused by smoking, which differed by provider type group (p = .03), with registered nurses most likely to agree. This belief also differed by age (p < .01). Among the < 25 and 25–34 age groups, 21.0% and 19.6%, respectively, agreed that nicotine causes cancer, while 34.8% and 34.0% of those in the 25–44 and 45 + groups agreed. Lastly, 42.8% agreed that nicotine in cigarettes is the substance that causes most of the CVD caused by cigarette smoking. This also differed by provider group (p < .01), with registered nurses most likely to agree (Fig 1).This also differed by age (p < .01), with those in the younger age groups less likely to agree compared with those in the older age groups.

**Fig 2:**
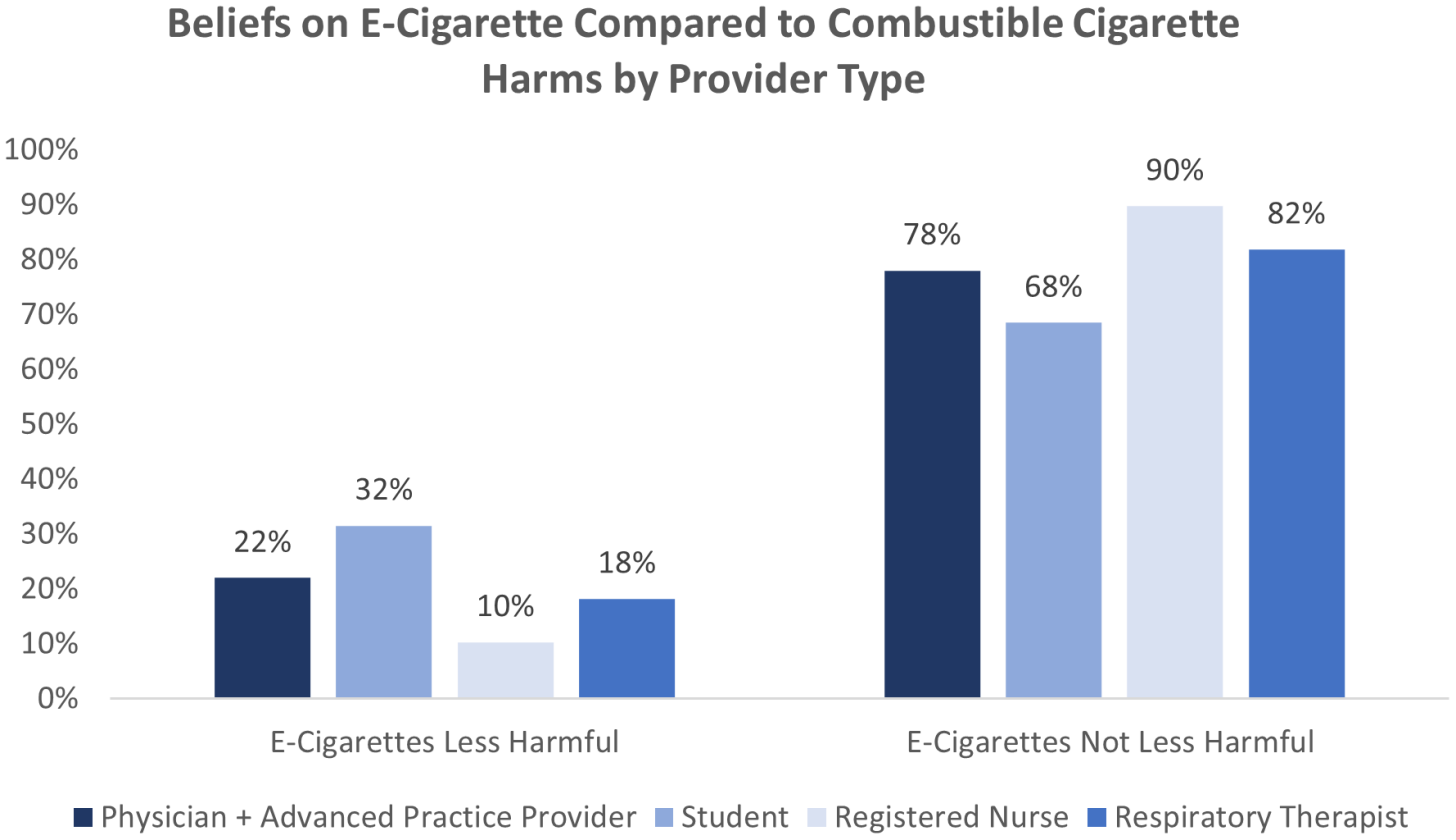
Perceptions of e-cigarette harm by provider type. Graph shows perception of e-cigarette harms by provider type.

The majority of participants (60.2%) believed e-cigarettes to be just as harmful or more harmful than combustible cigarettes, with 18.4% of respondents believing them to be much more harmful. Only 21.4% of participants believed e-cigarettes to be less harmful than combustible cigarettes (Fig 2). Those believing e-cigarettes to be less harmful than combustible cigarettes were more likely to be male (OR=2.11, 95% CI 1.32–3.37), a student (OR=1.88, 95% CI 1.20–2.94), and to disagree that nicotine is the main substance that causes cancer (OR=1.39, 95% CI 1.03–1.88) or CVD (OR=1.62, 95% CI 1.23–2.13). Receiving formal smoking cessation training, age, or having ever used tobacco were not associated with beliefs about e-cigarette harms.

## Discussion

In our single-center study of healthcare providers and students at an academic medical center, we found that healthcare professionals continue to hold inaccurate beliefs regarding the harms to health caused by nicotine. Although most healthcare providers in our sample correctly identified nicotine as the chemical that makes people want to smoke, many still incorrectly believe nicotine to be the cause of most of the cancer and CVD associated with cigarette smoking. These results are somewhat consistent with prior research that studied beliefs among physicians [13,14]. For instance, Steinberg et al. conducted a survey among a national sample of physicians and found that physicians believed that nicotine directly contributes to the development of cancer (80.5%), COPD (80.9%), and cardiovascular disease (83.2%) [14]. A subsequent study of physicians changed the wording of the question to be even more clear that the questions were about direct nicotine effects by asking if “nicotine, on its own, directly contributes to cancer” and other diseases. This study found that 82.4% of US physicians inaccurately agreed with this statement [13]. Our study, using slightly different wording in the questions, found a far lower proportion of health professionals holding inaccurate beliefs about the role of nicotine in causing cancer. Our data extends prior findings to other groups of health professionals and identified that registered nurses were the profession most likely to hold these inaccurate beliefs regarding the harms caused by nicotine. This finding may reflect gaps in education on nicotine and tobacco use, highlighting an opportunity to enhance nursing training and awareness in this area to ensure accurate understanding of nicotine-caused risks. In addition, our study noted that age was associated with beliefs about nicotine harms, with older providers more likely to hold inaccurate beliefs. Our understanding of nicotine and tobacco related harms has grown substantially since many providers were initially trained, highlighting the importance of continuing education on this topic, especially as new nicotine and tobacco products are rapidly entering the market.

Our study also identified that the majority of healthcare providers believed e-cigarettes to be equally or more harmful than traditional cigarettes. This finding‌‌ is consistent with several international reports, including a 2019 study in Poland, which revealed that over 80% of physicians believed e-cigarettes to be carcinogenic and increase the risk of CVD and COPD [34]. Similarly, a study conducted in China among 1028 respiratory medical staff (physicians and nurses) found that about 90% viewed e-cigarettes as generally harmful to health, with 60% disagreeing with their use as a smoking cessation tool [35]. The belief that e-cigarettes are just as harmful as traditional cigarettes has potential to influence healthcare providers’ likelihood of supporting e-cigarettes as a harm-reduction tool. Evidence suggests that tobacco users attempting to quit are more likely to utilize e-cigarettes than other methods and to achieve greater success in doing so [36]. Thus, discouraging e-cigarette use by smokers who may not have succeeded at quitting with other cessation methods may have unintended consequences.

Importantly, this study found a significant association between inaccurate beliefs about nicotine harms and e-cigarette harms. We found that participants who incorrectly believed nicotine to be the cause of cancer and CVD were also more likely to believe that e-cigarettes are equally, or more harmful than cigarettes. Conversely, participants with accurate beliefs regarding nicotine’s role, demonstrated a more accurate understanding of e-cigarette harms. To our knowledge, this is the first study to show a correlation between the healthcare providers’ beliefs about nicotine-caused harms and their beliefs regarding the relative harms from e-cigarette and cigarettes. Despite data showing that biomarker concentrations of toxicants in exclusive e-cigarette users are significantly lower when compared to exclusive cigarette smokers [37], the negative portrayal of e-cigarettes in the media often distorts or rejects the potential role in harm reduction strategies [38].

Lastly, widespread prevalence of inaccurate beliefs about the effects of nicotine among healthcare providers highlights the urgent need for targeted educational efforts. Incorrectly attributing cancer to nicotine can undermine healthcare providers’ confidence in recommending evidence-based treatments, such as pharmaceutical nicotine replacement therapy, for tobacco use disorder [38].

### Strengths and limitations

This study has a large sample size (n = 598) and a population containing a wide range of healthcare professional groups. The study also addresses an important knowledge gap by specifically examining the relationship between beliefs about nicotine and about e-cigarettes among healthcare providers.

Limitations include those commonly seen in survey research, such as self-report bias and lack of generalizability of the gathered data. Additionally, this study utilized convenience sampling technique, therefore the exact number of individuals who viewed the recruitment flyer is unknown. While all the data collection was completed at one institution; the participants had varying levels of training histories and were trained at a wide range of educational institutions. Another limitation of this study is the potential ambiguity in some of the questionnaire items related to nicotine, which may have influenced participant responses. However, we believe the data remain reliable and accurate given the consistency of responses observed across the dataset and given prior studies demonstrating similar results with varying question wording.

## Conclusions

This study demonstrates common inaccurate beliefs regarding the harms of nicotine among healthcare providers. While most healthcare providers correctly identified nicotine as the substance that makes people want to smoke, a considerable proportion mistakenly attributed most of the cancer and CVD risk associated with tobacco use to nicotine. Furthermore, our findings revealed a strong association between inaccurate beliefs about nicotine’s harms and the belief that e-cigarettes are equally or more harmful than combustible cigarettes. These results highlight the importance of targeted educational initiatives to address widespread misconceptions about nicotine’s role in addiction versus causing diseases like cancer. By clarifying the distinct harms associated with nicotine, tobacco combustion products, and e-cigarettes, these interventions can equip healthcare providers to deliver evidence-based counseling on smoking cessation and harm reduction strategies. Future efforts should prioritize training that emphasizes the relative risks of various nicotine delivery systems, enabling providers to guide patients more effectively in their efforts to reduce tobacco-related harm.
